# Shifting communities after­­ typhoon damage on an upper mesophotic reef in Okinawa, Japan

**DOI:** 10.7717/peerj.3573

**Published:** 2017-08-18

**Authors:** Kristine N. White, David K. Weinstein, Taku Ohara, Vianney Denis, Javier Montenegro, James D. Reimer

**Affiliations:** 1 Department of Biology, The University of Tampa, Tampa, FL, United States of America; 2 Graduate School of Engineering and Science, University of the Ryukyus, Nishihara, Okinawa, Japan; 3 Benthos Divers, Onna, Okinawa, Japan; 4 Institute of Oceanography, National Taiwan University, Taipei, Taiwan; 5 Tropical Biosphere Research Center, University of the Ryukyus, Nishihara, Okinawa, Japan

**Keywords:** Mesophotic, Succession, Coral reef, *Pachyseris*, Japan, Typhoon recovery, Shifting communities

## Abstract

Very few studies have been conducted on the long-term effects of typhoon damage on mesophotic coral reefs. This study investigates the long-term community dynamics of damage from Typhoon 17 (Jelawat) in 2012 on the coral community of the upper mesophotic Ryugu Reef in Okinawa, Japan. A shift from foliose to bushy coral morphologies between December 2012 and August 2015 was documented, especially on the area of the reef that was previously recorded to be poor in scleractinian genera diversity and dominated by foliose corals. Comparatively, an area with higher diversity of scleractinian coral genera was observed to be less affected by typhoon damage with more stable community structure due to less change in dominant coral morphologies. Despite some changes in the composition of dominant genera, the generally high coverage of the mesophotic coral community is facilitating the recovery of Ryugu Reef after typhoon damage.

## Introduction

Scleractinian corals are the primary architects of reef ecosystems and the major contributors to reef rugosity, a fundamental parameter for the resilience of the ecosystem after a disturbance (Graham et al., 2015). Therefore, documenting the extent of damage to corals after perturbations is key to understanding the potential trajectory of recovery. To quantify the shifts in functional composition of coral reefs after environmental and anthropogenic disturbances, Darling et al. (2012) determined coral life history strategies based on several traits, including growth form, reproductive mode, and fecundity. Shifts to stress-tolerant, generalist and weedy species after disturbances have been documented in both the Caribbean (Alvarez-Filip et al., 2009; Alvarez-Filip et al., 2011) and the Indo-Pacific (McClanahan et al., 2007; Rachello-Dolmen & Cleary, 2007). Recruitment of coral larvae can be an important factor in the recovery of a coral reef (Pearson, 1981), but plays a lesser role if there is regrowth from surviving coral colonies and/or if fragmentation occurs (Hughes, 1985). Shallow areas with few local survivors are most likely dependent on recruitment and show low rates of recovery, whereas areas with many survivors often show rapid recovery due to regrowth of remnant colonies (Connell, Hughes & Wallace, 1997).

The recovery of coral reefs after disturbances is usually measured by changes in coral cover, abundance, species composition, and/or diversity (Davis, 1982; Connell, Hughes & Wallace, 1997; Graham et al., 2015). The type of disturbance, original species composition, reef complexity (rugosity), and depth are all thought to be responsible for the wide variation in patterns of recovery. For example, a 95-year study of Dry Tortugas Reef in Florida demonstrated a relatively constant abundance of coral and other benthic organisms, despite changes in species composition and coral reef structure (Davis, 1982). However, if coral cover is significantly reduced after a disturbance, the reef will likely undergo a shift in the composition of coral species to the coral species that survived the disturbance (Connell, Hughes & Wallace, 1997), or to other taxa, such as fleshy macroalgae. For example, phase shifts from scleractinian coral dominance to fleshy macroalgae were observed after multiple storm and anthropogenic disturbances on Jamaican reefs over 40 years (Hughes, 1994).

Reports measuring coral reef recovery after storm disturbances based on coral cover on shallow reefs vary in time from two to 15 years, with little attention paid to species composition (Pearson, 1981; Platt & Connell, 2003; Gardner et al., 2005). Ecological succession (the replacement of early species with late species; Cowles, 1899) occurs following disturbances, and community recovery processes are affected by different types of disturbances that create the opportunity for a change in succession (Platt & Connell, 2003). Storms often result in various successional states or phase shifts, for which the capacity to return to the original condition has been intensively debated (Dudgeon et al., 2010; Bruno, 2014; Dixon et al., 2015). Coral cover throughout Caribbean coral reefs decreased by an average of 17% after hurricanes between 1980 and 2001, with no further coral loss in the year following a disturbance and no evidence of recovery eight years after a disturbance (Gardner et al., 2005). Gardner et al. (2005) suggested that the lack of recovery was a result of other stressors such as sedimentation or eutrophication impacting shallow reefs and that exposure to storms makes coral reefs less susceptible to storms in the future due to the recovery of more tolerant species.

Most coral reef storm recovery studies have been conducted on relatively shallow Caribbean reefs (e.g., Stoddart, 1974; Shinn, 1976; Pearson, 1981), but more work is needed to understand storm recovery on Indo-Pacific reefs in general and mesophotic reefs in particular. In one example from the Indo-Pacific, coral cover on a 6–12 m tabulate *Acropora* reef in the Coral Sea declined from 80% to less than 10% after several storms over a two year period, leaving only encrusting and robust coral species (Halford et al., 2004). This was followed by coral cover increasing exponentially 5–9 years after the storm disturbances, with the reef having recovered to pre-storm levels after 11 years, albeit with a shift from branching to tabulate coral morphology (Halford et al., 2004). In another example, Nozawa, Lin & Chung (2013) examined coral recruitment at 5 and 15 m depths in Taiwan, and concluded that shallower coral reefs with more pocilloporid and poritid recruits were more influenced by post-settlement processes compared to deeper reefs that have more acroporid recruits and were more affected by pre-settlement processes.

Unlike their shallow-water counterparts, there is very little information available documenting recovery of mesophotic coral reefs (benthic communities including hermatypic zooxanthellate corals at 30–150 m depths (Baker, Puglise & Harris, 2016)) after storm disturbances. Although it has often been assumed that mesophotic reefs are relatively protected from storm damage (Bongaerts et al., 2010), recent studies have shown this is not the case (Bongaerts, Muir & Bridge, 2013; White et al., 2013). The impact of storms on patterns of benthic communities documented on large horizontal scales can help to determine upper mesophotic shelf edge (30–60 m) coral development (Smith et al., 2016). Depending on the topography and distance between shallow and deep reefs, storms can damage corals on low-angle slopes as a result of coral debris or sediment transported down the reef slope compared to high-angle slopes (Bongaerts et al., 2010); whereas other low-angle upper mesophotic reefs do not appear to be impacted by terrestrial sediment transport (Weinstein, Klaus & Smith, 2015). Growth rates of different coral species may also be a factor in recovery processes (Bongaerts et al., 2015). Studies suggest that upper mesophotic coral species in the Caribbean have comparatively slower growth rates than shallow water coral species (Dustan, 1975; Hughes & Jackson, 1985; Leichter & Genovese, 2006; Weinstein et al., 2016). However, Bongaerts et al. (2015) reported that growth rates of some coral species at lower mesophotic depths (60–150 m) may be similar to observed rates in shallow water.

One of the few ecological studies monitoring the direct impact of storm damage on a mesophotic reef system is from Okinawa, Japan. Ohara et al. (2013) described Ryugu Reef as an upper mesophotic reef with a large, nearly monospecific stand of *Pachyseris foliosa* from 32 to 45 m. Heading deeper, away from shore, Ryugu Reef transitions on a low-angle slope from rubble with some corals (Fungiidae) at 21 m; to a high diversity of corals at 26 m; a *Pachyseris-* dominated area at 31 m; and finally to sand at 42 m. The center of Typhoon 17 (Jelawat), the strongest typhoon ever recorded to hit Okinawa-jima Island, with wave heights up to 12 m, passed within 30 km of Ryugu Reef on 29 September 2012 (Ohara et al., 2013; White et al., 2013). Analyses of coral species composition and morpho-functional groups on Ryugu Reef before and after Typhoon 17 suggested that the highly diverse areas were less susceptible to typhoon damage (White et al., 2013). White et al. (2013) also theorized that Ryugu Reef would be resilient because of a high likelihood of recovery based on only slight changes in functional groups after the typhoon. Despite such speculation, little is known regarding long-term recovery of mesophotic reef systems following major storm events.

Previous studies suggest that coral communities recover from storm disturbances via succession (Gardner et al., 2005; McClanahan et al., 2007; Rachello-Dolmen & Cleary, 2007; Alvarez-Filip et al., 2009; Alvarez-Filip et al., 2011; Hughes et al., 2012; Smith et al., 2016), with dominant coral genera shifting until the reef can mature and possibly return to initial conditions (Dudgeon et al., 2010; Bruno, 2014). Halford et al. (2004) observed exponential coral growth on the southern Great Barrier Reef 5–9 years after storms. Signs of recovery were documented on the Great Barrier Reef as early as 24–35 months after Tropical Cyclone Yasi in 2011, with evidence of regrowth of branching corals on King Reef (Perry et al., 2014) and an average 4% increase in coral cover on 19 reefs in the Great Barrier Reef Marine Preserve (Beeden et al., 2015). Combined, these results suggest that Indo-Pacific reefs’ successional changes may occur only a few years after a storm event. Thus, the objectives of this study include documenting the initial recovery of an upper mesophotic coral reef (Ryugu Reef) over a four-year period (2012–2015), and describing resulting changes in scleractinian coral communities based on functional morphology and species composition. This study provides the first in-depth record of an upper mesophotic reef shifting communities based on functional group changes after a storm disturbance and offers insight into the implications of this shift in the recovery of mesophotic coral reefs.

## Materials and Methods

**Figure 1:**
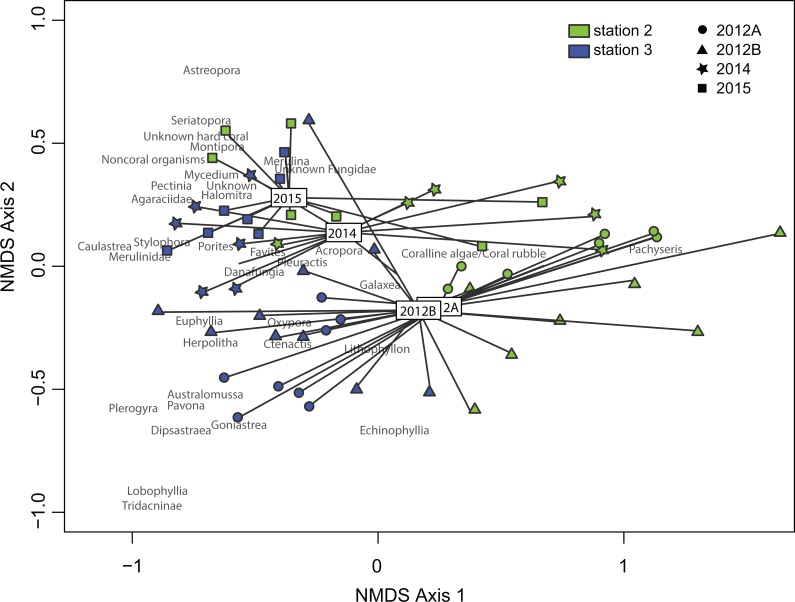
nMDS genus data. nMDS genus data with stress value of 0.16. Station factor (Bray-Curtis dissimilarities test, *R*^2^ = 0.41, *p* < 0.001); temporal factor (Bray-Curtis dissimilarities test, *R*^2^ = 0.19, *p* < 0.001). 2012A refers to pre-typhoon data and 2012B refers to post-typhoon data, both from White et al. (2013).

Random line transects were surveyed at Ryugu Reef (see map of stations in Fig. 1B; White et al., 2013) at seven locations near station 2 (30–32 m) and seven locations near station 3 (26–30 m). Adjacent 4 × 6 cm^2^ photographs (12–79 per transect) were taken along a 10-m tape for each line transect 1.5 years after Typhoon 17 (designated ‘2014’ dataset) and 2.5–3 years after the typhoon (designated ‘2015’ dataset). Scleractinian coral (+ one *Millepora* sp., hereafter ‘coral’) operational taxonomic units (OTUs) were identified in a similar manner as in White et al. (2013), following Hoeksema (1989) and Gittenberger, Reijnen & Hoeksema (2011) for Fungiidae, Huang et al. (2016) for Lobophylliidae, Huang et al. (2014) for Merulinidae, Diploastraeidae and Montastraeidae, and Veron (2000) for all remaining corals. Scientific binomens were checked for validity in the World Register of Marine Species (WoRMS; Hoeksema & Cairns, 2015; accessed May 2, 2017). Post-typhoon data from 2012 (White et al., 2013) were combined with the newest dataset. Many mesophotic coral species at Ryugu, particularly those within genera *Acropora* (see Wallace, 1999)*, Galaxea*, and *Montipora*, were very hard to conclusively identify to species level. Therefore, changes in the community were investigated at the genus-level. Bray-Curtis dissimilarities were calculated on raw data by comparing each transect to every other transect, and visualized using non-metric multidimensional scaling (nMDS). Time-period centroids (spatial median) were overlaid to visualize temporal dynamics. Differences in the composition of the benthic assemblage between stations, and between temporal dynamics within each station were tested using permutational multivariate analysis of variance (ADONIS).

Each coral OTU was described following similar methods to those described in Denis et al. (2013) and White et al. (2013). Corals were assigned to one or more of eight morphological groups as in White et al. (2013), following growth form information found in Wallace (1999), Veron (2000), Pillay (2002) and Bellwood et al. (2004) as well as by confirming morphologies from *in situ* photographs. OTU categories (massive/submassive, encrusting, laminar/foliose, columnar, plate-like, bushy, arborescent and unattached morphologies) are provided in Table S1. Often, colonies presented more than one growth form in the field, and these OTUs were therefore assigned to two growth forms. Community-level Weighted Means (CWM) of trait values (Lavorel et al., 2008) were computed for each transect, and standardized to determine the relative contribution of each morpho-functional group to the coral assemblage (removing all non-coral OTUs). Contribution of a given morpho-functional group was summarized for each station/period sampled and compared among years using Kruskal-Wallis tests followed by Conover’s multiple comparison tests.

All data were analyzed in R v3.2.3 (R Core Team, 2014) using the packages Vegan (Oksanen et al., 2016) and FD (Laliberté & Legendre, 2010; Laliberté, Legendre & Shipley, 2014).

## Results

**Figure 2:**
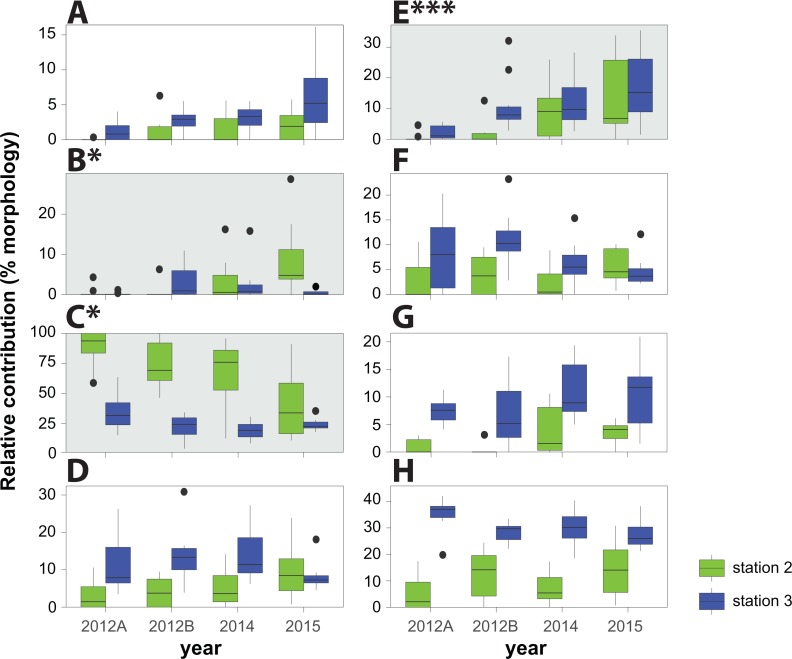
Community weight mean (CWM) of trait values. Community weight mean (CWM) of trait values representing the contribution (%) of corals representing each given morphology. Significant results are shaded with a grey background (Kruskal-Wallis/Conover tests, * *p* < 0.05; *** *p* < 0.01). 2012A refers to pre-typhoon data and 2012B refers to post-typhoon data, both from White et al. (2013). (A) Arborescent; (B) Plate-like; (C) Laminar & Foliose; (D) Massive & Submassive; (E) Bushy; (F) Columnar; (G) Unattached; (H) Encrusting.

Relative live coral cover increased at each station by 5% between 2012 and 2014 and by another 6% between 2014 and 2015 (Table 1). Significant changes in cover of coral genera occurred over time at both stations 2 and 3 (ADONIS test, *R*^2^ = 0.19, *p* < 0.001) (Fig. 1). However, variation between stations (ADONIS test, *R*^2^ = 0.41, *p* < 0.001) was greater than among years (Fig. 1). Station 3 showed minor changes in cover of coral genera between 2014 and 2015 after a major shift in coral assemblage between 2012 and 2014. Station 2 showed diversification of coral genera in the three years after Typhoon 17 (Jelawat) with a decrease in *Pachyseris* cover and an increase in several other genera (Table 1). An increase in cover of bushy corals (*Acropora*, *Porites*, *Seriatopora*, *Stylophora*) was significant (Kruskal-Wallis/Conover tests, *p* < 0.01) at station 3 as of 2015, compared to 2012 before the typhoon (Fig. 2). Significant coral cover changes at station 2 as of 2015 included a decrease of foliose corals, compared to 2012 before the typhoon (Kruskal-Wallis/Conover test, *p* < 0.05), an increase of bushy corals (Kruskal-Wallis/Conover test, *p* < 0.01), and an increase in some plate-like corals (*p* < 0.05) (Fig. 2). A major factor in the decrease of foliose coral cover at station 2 was the continuous decrease of *Pachyseris* from 2012 (post-typhoon) to 2015 (Table 1).

**Table 1:** Relative percent coral. Relative percent cover of coral genera, coral rubble/coralline algae, and pavement at stations 2 and 3 over time after Typhoon Jelawat, 2012–2015.

Taxonomic units	Station 2 (%)			Station 3 (%)		
	December 2012	April 2014	March 2015	December 2012	April 2014	August 2015
*Acropora*	<1	9	17	2	11	18
*Agaraciidae*	0	<1	<1	0	0	0
*Astreopora*	0	0	<1	0	<1	<1
*Australomussa*	0	0	<1	<1	<1	0
*Caulastrea*	0	0	0	0	<1	<1
*Ctenactis*	<1	<1	<1	2	2	3
*Danafungia*	<1	2	2	2	3	2
*Dipsastraea*	0	0	0	<1	0	<1
*Echinophyllia*	<1	<1	<1	4	<1	<1
*Euphyllia*	0	0	<1	<1	<1	<1
*Favites*	0	0	0	<1	0	0
*Galaxea*	2	5	10	10	12	8
*Halomitra*	0	0	<1	<1	<1	0
*Herpolitha*	0	<1	<1	1	1	1
*Lithophyllon*	2	2	1	19	10	12
*Lobophyllia*	0	0	0	<1	0	0
*Merulina*	0	0	<1	0	<1	1
*Montipora*	0	<1	2	0	<1	4
*Mycedium*	0	<1	1	<1	1	1
*Oxypora*	0	<1	<1	0	<1	0
*Pachyseris*	58	49	34	3	4	4
*Pavona*	0	0	<1	4	<1	2
*Pectinia*	0	0	<1	0	<1	<1
*Pectiniidae*	0	0	0	0	3	0
*Pleuractis*	<1	1	1	1	3	3
*Porites*	0	<1	<1	<1	<1	<1
*Seriatopora*	0	<1	<1	0	<1	0
*Stylophora*	0	0	<1	0	<1	<1
*Turbinaria*	0	0	0	<1	0	0
Coral rubble/coralline algae	37	21	7	49	32	22
Pavement	N/A	11	19	N/A	12	16
Unknown live coral	0	<1	<1	<1	1	1
Total live coral cover	63	68	74	51	56	62

Percent cover of coral rubble/coralline algae decreased from 31% to 27% at station 2 between 2012b and 2014 from 27% to 7% between 2014 and 2015 (Table 1). This followed a 29% increase in coral rubble/coralline algae directly after typhoon Jelawat (White et al., 2013). At station 3, coral rubble/coralline algae decreased from 49% to 32% between 2012b and 2014 and from 32% to 22% between 2014 and 2015.

## Discussion

**Figure 3:**
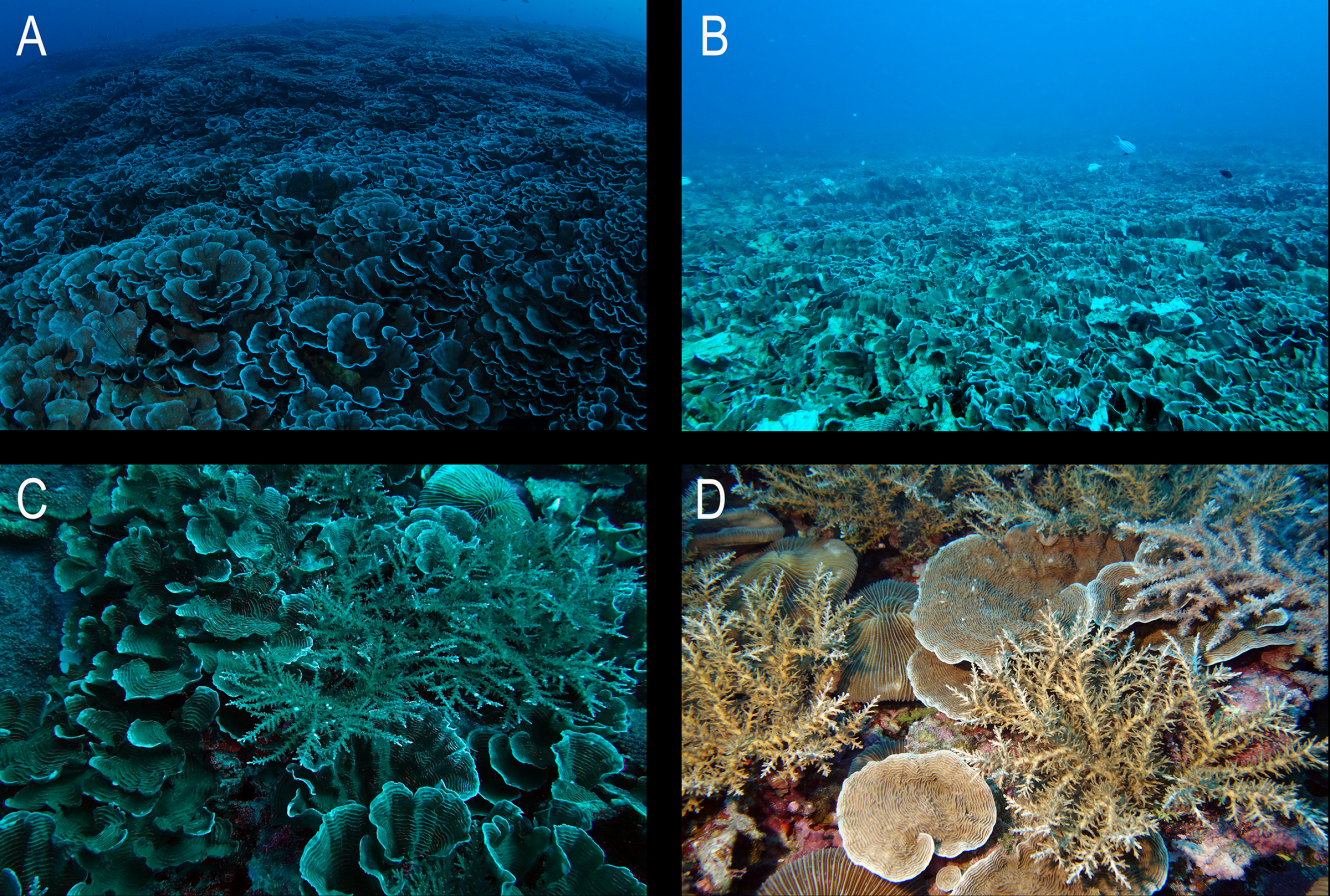
Station 2. Example of community shift from foliose to bushy coral genera on Ryugu Reef at station 2 (30–32 m) from (A) 2012 (pre-typhoon); (B) 2012 (post-typhoon); (C) 2014; and (D) 2015.

Data are sparse on the recolonization of mesophotic reefs. The diversification of mesophotic coral reef organisms after a disturbance may occur as a result of horizontal connectivity with other mesophotic reefs or vertical connectivity with shallow water reefs (Kahng, Copus & Wagner, 2014). Species with wide depth ranges are more likely to benefit from vertical connectivity, while horizontal connectivity would most likely affect only the acquisition of symbiotic zooxanthellae for species that acquire *Symbiodinium* from the water column (Bongaerts et al., 2010; Kahng, Copus & Wagner, 2014). Over a short time, it is likely that a storm disturbance would open up space in the area previously documented with nearly 100% *Pachyseris* coverage (Ohara et al., 2013), allowing more opportunistic species a chance to prosper. Station 2 showed a diversification of coral species in the three years after Typhoon 17 (Jelawat) created vacant ecological niches (Table 1). *Pachyseris* (foliose morphology) cover decreased immediately following the typhoon (White et al., 2013), allowing the increased growth of arborescent, bushy, columnar, massive, and encrusting morphologies (Fig. 3), although significant increases were only seen in bushy genera at this station (Kruskal-Wallis/Conover test, *p* < 0.01). At least six of the ‘massive’ genera (*Astreopora*, *Caulastrea*, *Favites*, *Galaxea*, *Goniastrea*, *Plerogyra*) have been noted to be ‘stress-tolerant’ and four ‘weedy’ genera (fast growing with high turnover) are known to do well under disturbance (*Goniastrea, Porites*, *Seriatopora*, *Stylophora*) (Darling et al., 2012). However, the dominant genus (*Pachyseris*) on Ryugu Reef is a ‘generalist’ (Darling et al., 2012). It appears that Typhoon Jelawat created the opportunity for genera aside from *Pachyseris* to increase coverage, and these genera could possibly consist of opportunistic species at upper mesophotic depths. Similarly, areas of the Great Barrier Reef recovered at different rates after disturbances, with the fastest recovery rates occurring when more space was available as a result of reduced coral cover (Graham et al., 2014). At Ryugu Reef, coral rubble cover decreased from the post-typhoon survey of 2012 (Table 1), and *Pachyseris* coral cover continually decreased in the four years post-typhoon. Following damages from the typhoon, diversification of the area previously dominated by *Pachyseris* corals occurred, with an increase in cover of genera such as *Seriatopora* and *Galaxea*. It is possible that colonies of these genera were previously present but in much smaller numbers than *Pachyseris* or that these genera contain opportunistic species.

Immediately after Typhoon 17, little change in coral community assemblage was evident at the shallower station 3, suggesting that the diverse/complex community was more resistant due to lower impact on individual genera than the impact on the *Pachyseris*-dominated community (White et al., 2013). At station 3, corals recovered with progressive recolonization of the available substrate via surviving corals between 2012 (post-typhoon) and 2015 (Table 1). Cover of *Acropora*, *Ctenactis*, *Danafungia, Porites*, *Seriatopora,* and *Stylophora* increased, suggesting that bushy and unattached coral genera did better than the foliose genera (*Montipora*, *Mycedium*, *Oxypora*, *Pachyseris*) that stabilized with no significant increase or decrease in cover at station 3 (Table 1).

Major shifts in functional groups were evident at both stations 2 and 3 post-typhoon (Fig. 1). Foliose genera decreased at stations 2 and 3 after the typhoon, with a significant decrease as of 2015 at station 2 (Kruskal-Wallis/Conover test, *p* < 0.05). These results are similar to a previous shallow-water study on the Great Barrier Reef in which foliose coral colonies declined drastically after a storm event (Perry et al., 2014). Bushy coral genera increased at both Ryugu stations post-typhoon (Kruskal-Wallis/Conover test, *p* < 0.01). The functional shift shows that beyond the apparent recovery of Ryugu Reef, the trajectory followed could lead ultimately to a fundamentally different coral community, as previously reported from the Great Barrier Reef (Harmelin-Vivien, 1994). However, maturation of the coral assemblage with time may lead to the return of a state dominated by generalist *Pachyseris* corals (Darling et al., 2012). Continued monitoring of Ryugu Reef will determine whether this community will return to the initial state recorded prior to Typhoon 17 or if eventually the ecosystem might reach stability in an alternative configuration.

White et al. (2013) hypothesized that mesophotic reefs such as Ryugu would be resilient and recover quickly after disturbance to the original conditions of the reef. The current data do not support this hypothesis as a shift in community structure to more bushy and columnar morphologies and less foliose morphologies was observed. The large *Pachyseris* stand at station 2 was heavily damaged after Typhoon 17 (Jelawat) in 2012, but showed signs of recovery in 2015 with only slight diversification of the coral community. This fast recovery, combined with behavioral characteristics, may enable *Pachyseris* spp. to outcompete other coral genera at upper mesophotic depths, allowing Ryugu Reef to return to the initial, pre-typhoon coral assemblage. However, if Ryugu Reef coral genera are limited by their growth rates as many other mesophotic coral genera are (Dustan, 1975; Hughes & Jackson, 1985; Leichter & Genovese, 2006; Weinstein et al., 2016), it is more likely that there will be a permanent shift with increasing presence of weedy or stress-tolerant species (McClanahan et al., 2007; Rachello-Dolmen & Cleary, 2007; Alvarez-Filip et al., 2009; Hughes et al., 2012). While the dominant genera are changing on Ryugu Reef, there were no new species recorded, suggesting the regrowth of damaged corals or local recruitment. Examinations of shallow *Acropora* corals around Okinawa Main Island (Shinzato et al., 2015) and on the Great Barrier Reef after Cyclone Yasi (Lukoschek et al., 2013) both suggest the importance of local recruitment for replenishment of *Acropora* species, and such research remains to be conducted on corals of deeper reefs such as Ryugu Reef. The high live coral cover of Ryugu Reef suggests that although mesophotic reefs may be affected by large storms, recovery may be facilitated by the relative stability of the mesophotic zone.

##  Supplemental Information

10.7717/peerj.3573/supp-1 Supplemental Information 1 Data for statistical analyses

10.7717/peerj.3573/supp-2 Table S1 Supplementary Material Table S1: Trait data Morpho-functional groups of observed Scleractinia operational taxonomic units at Ryugu, Okinawa, Japan, 2012–2015.

## Additional Information and Declarations

### Data Availability

The following information was supplied regarding data availability: Data used for statistical analyses is uploaded as a Supplemental File.
